# Feasibility of virtual starshot analysis providing submillimeter radiation isocenter accuracy: A long‐term multi‐institutional analysis

**DOI:** 10.1002/acm2.12715

**Published:** 2019-09-09

**Authors:** Yuichi Akino, Masateru Fujiwara, Hirokazu Mizuno, Hiroya Shiomi, Akari Kaneko, Fumiaki Isohashi, Yuji Seo, Osamu Suzuki, Keisuke Otani, Keisuke Tamari, Kazuhiko Ogawa

**Affiliations:** ^1^ Oncology Center Osaka University Hospital Suita Osaka 565‐0871 Japan; ^2^ Suita Tokushukai Hospital Suita Osaka 565‐0814 Japan; ^3^ Division of Health Sciences Osaka University Graduate School of Medicine Suita Osaka 565‐0871 Japan; ^4^ Department of Radiation Oncology Osaka University Graduate School of Medicine Suita Osaka 565‐0871 Japan; ^5^ Department of Carbon Ion Radiotherapy Osaka University Graduate School of Medicine Suita Osaka 565‐0871 Japan

**Keywords:** IGRT, radiation isocenter, stereotactic radiotherapy, Winston–Lutz test

## Abstract

**Purpose:**

We developed a technique to calculate the offset between room lasers and the radiation isocenter using a digital Winston–Lutz (WL) test with a starshot technique. We have performed isocenter localization quality assurance (QA) with submillimeter accuracy for a long period. Here we evaluated the feasibility and accuracy of this virtual starshot (VS) analysis for isocenter localization QA.

**Methods:**

A 6‐MV photon beam with a square multileaf collimator field was used to irradiate a WL sphere positioned at the intersection of the room lasers. Images were acquired using an electronic portal imaging device. A four‐field WL test was performed, and the path of each beam was calculated from the offset between the beam and sphere. Virtual starshot analysis was used to analyze the radiation isocenter, which calculates the center of the beam paths by using a least‐squares method, similar to the starshot analysis. Then, eight coplanar and 12 noncoplanar beams were irradiated to evaluate isocenter localization accuracy.

**Results:**

Several VS analyses, using different WL spheres, were performed at three institutions, and the calculated accuracies were within 0.1 mm at all institutions. Long‐term analysis showed that the isocenter localization accuracy was appropriately managed with three‐dimensional accuracy within ± 0.5 mm for 90 months after the first laser adjustments. The offset between each beam and the room laser was within 0.6 mm and within 1.0 mm for eight coplanar and 12 noncoplanar beams, respectively, for 90 months. Cone‐beam computed tomography images, acquired after verification beams, showed that the offset between the radiation isocenter and the imaging center was within 0.66 mm for 90 months*.* The isocenter localization accuracy within 1 mm was kept for long period at other four institutions.

**Conclusions:**

Long‐term analysis showed the feasibility of VS analysis for isocenter localization QA, including room laser re‐alignment, noncoplanar irradiation verification, and image guidance accuracy.

## 1 INTRODUCTION

Single‐fraction stereotactic radiosurgery (SRS) and fractionated stereotactic radiotherapy (SRT) have shown excellent clinical outcomes for metastatic brain tumors.^1^, ^2^, ^3^ Linear accelerator‐based SRS and SRT have been performed with circular cones mounted on the linac head or multileaf collimators (MLC). Recently, simultaneous irradiations to multiple brain metastases have been enabled.^4^ Such advanced techniques require high accuracy in mechanical structures, image‐guided radiotherapy (IGRT), patient immobilization, and beam modeling of treatment planning systems (TPS) for small‐field dosimetry.^5^, ^6^ The American Association of Physicists in Medicine, Task Group 142, recommends an accuracy of < ±1 mm of the localizing lasers as well as the imaging and treatment coordinate coincidence.^7^ One of the major techniques for evaluating isocenter localization is the Winston–Lutz (WL) test, which uses a small sphere and a piece of film and evaluates the targeting accuracy of irradiation by checking whether the sphere is projected inside the radiation field.^8^ Although the original WL test used four oblique gantry angles, with and without couch rotation, various patterns have been reported 9, 10. This technique is useful for quality assurance (QA) of the SRS for brain tumors, which often use noncoplanar beams to achieve highly conformal dose distribution. Recently, WL tests using an electronic portal imaging device (EPID) have been reported.^10^, ^11^, ^12^ The EPID generates digital image data and enables quick and quantitative evaluation of the WL tests.

Several studies have reported an algorithm that calculates the isocenter location with submillimeter accuracy; others have stated that their digital WL tests can be used to re‐align the room lasers to target the radiation isocenter.^11^, ^12^, ^13^, ^14^, ^15^ Even if the offset between the room lasers and radiation isocenter is calculated with submillimeter accuracy, however, it is very difficult to adjust the room laser localization to the radiation isocenter with submillimeter accuracy because the translations, rotations, and tilts of the lasers equipped in a radiotherapy treatment room are usually controlled manually. We developed an analysis technique to calculate the offset between the room lasers and radiation isocenter using a WL test and a starshot technique. Although previous studies^9^, ^12^ have shown techniques to localize the isocenter accuracy using the WL test, they did not report on the routine use of their techniques for long term. Our institution used this “virtual starshot” analysis (VS analysis) as routine isocenter localization QA for a long period. In addition, we collected the VS analysis data from multiple institutions where this method was used periodically. Here we analyzed the long‐term data of multiple institutions to evaluate the feasibility and accuracy of VS analysis.

## 2 METHODS

### 2.1 Image acquisitions

**Figure 1:**
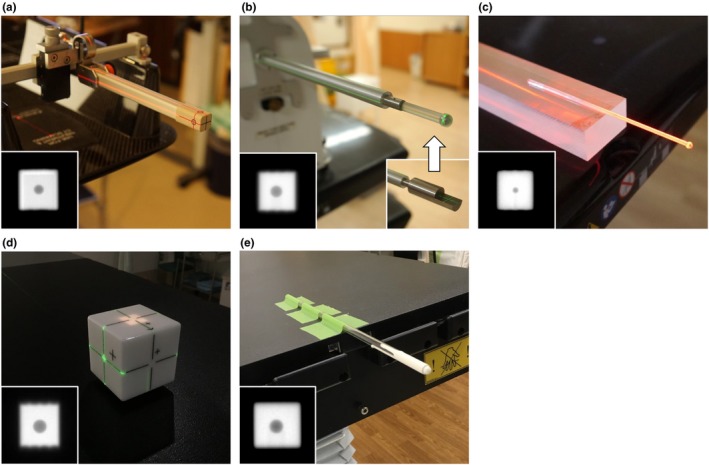
Winston–Lutz (WL) rods used at different institutions and example images acquired with these spheres. The institutions using each rod are listed in bottom right. (a) Institution *A*, (b) institutions *B* and *F*, (c) institution *C*, (d) institution *D*, and (e) institution *E*.

In this study, images for the WL test data were collected from six institutions (*A–F*). VS analysis was routinely conducted at institution *A* and *C–F*. The linacs and spheres used for the WL test are listed in Table 1. The external visible marks on the sphere phantoms were aligned with the room lasers. Here, two centers were defined: the sphere center and radiation isocenter. Images were acquired using an EPID. Most institutions used the field size of 20 × 20 mm^2^, whereas only institution *A* used the size of 10 × 10 mm^2^ because this institution conducted brain SRS using 2.5‐mm MLC leaf width. Because the MLC's nominal positional accuracy was better than that of the jaws, the field size was defined by MLC. The collimator angle was set to 90° for all beams to minimize the effects of gravity, although patients were treated with various collimator angles optimized for each plan. Figure 1(b) shows a WL test rod containing a 5‐mm tungsten sphere (Taisei Medical, Osaka, Japan), which was manufactured as an attachment for Iso‐Align (CIVCO Medical Solutions, Orange City, IA). First, a stainless rod with cross‐hair lines (shown in the right‐bottom window) was used to adjust the position to the room laser, and then the rod was exchanged with the WL test rod. Figures 1(c) and 1(e) shows hand‐made acrylic rods with a 3‐mm steel sphere and a 5‐mm tungsten sphere, respectively. The spheres were painted white to improve the visibility of the laser projected on it.

**Table 1:** Linear accelerators and spheres used for Winston–Lutz test.

Institutions	A	B	C	D	E	F
Linac	TB STx	TB	TB	CLINAC iX	CLINAC iX	Oncor
MLC	HD120	Millennium120	Millennium120	Millennium120	Millennium120	160MLC
EPID	aS1000	aS1200	aS1200	aS1000	aS1000	OPTIVUE 1000
Resolution	0.392 mm	0.336 mm	0.336 mm	0.392 mm	0.392 mm	0.400 mm
SSD	1500 mm	1500 mm	1500 mm	1500 mm	1500 mm	1480 mm
Lutz Sphere	BrainLAB	Taisei Medical	Hand‐made	SNC	Hand‐made	Taisei Medical

TB, TrueBeam (Varian Medical Systems, Palo Alto, CA); Oncor Impression Plus (Siemens, Erlangen, Germany); BrainLAB (Munich, Germany), a frameless SRS QA target pointer; Taisei Medical (Osaka, Japan), a custom‐made rod containing 5‐mm sphere; SNC, WLQA (Sun Nuclear Corp., Melbourne, FL).

### 2.2 Winston–Lutz test

**Figure 2:**
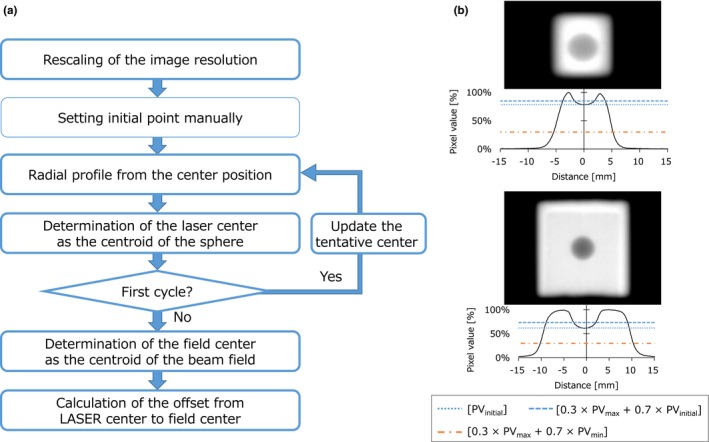
(a) A flowchart of the Winston–Lutz analysis. Boxes with the thick frame represent the steps automatically calculated in the software. (b) Schemes of the Winston–Lutz analysis for the multileaf collimators (MLC) field sizes of (upper) 10 × 10 mm^2^ and (lower) 20 × 20 mm^2^. LAT, lateral; Vert, vertical; Long, longitudinal.

The acquired images were analyzed using Akilles RT software (RADLab, Osaka, Japan). A flowchart and calculation algorithm of the WL analysis are shown in Fig. 2. First, the pixel size recorded in the DICOM file header was corrected by source‐imager‐distance, and the images were resized to twice as large using linear interpolation to detect the sphere and field edges smoothly; the pixel size was 0.131 mm at the isocenter plane after correction for EPID with original pixel size of 0.392 mm. This process may lead to uncertainty of the analysis, although the impact is smaller than pixel size. To determine a threshold for detecting the sphere, an initial point was manually set. From the initial point, the pixel values were scanned radially every 2° and line profiles were acquired. To obtain the contour of the sphere, a threshold of [0.3 × PV_max_ + 0.7 × PV_initial_] was used. Here, PV_max_ and PV_initial_ represent the global maximum pixel value and initial pixel value, respectively. The sphere centroid was defined at the intersection of the room lasers. To reduce the uncertainty owing to the manual procedure of setting initial point, the calculated sphere center was set as the initial point, and the analysis was repeated. The contour of the radiation field was also extracted from the radial profiles using a threshold value of [0.3 × PV_max_ + 0.7 × PV_min_] and the radiation field centroid was calculated. Here, PV_min_ represents global minimum pixel value. Then, the offsets between the radiation field and sphere represent the relative displacements of the radiation beam from the room laser (*δ*_Beam, Laser_).

### 2.3 Virtual starshot analysis

**Figure 3:**
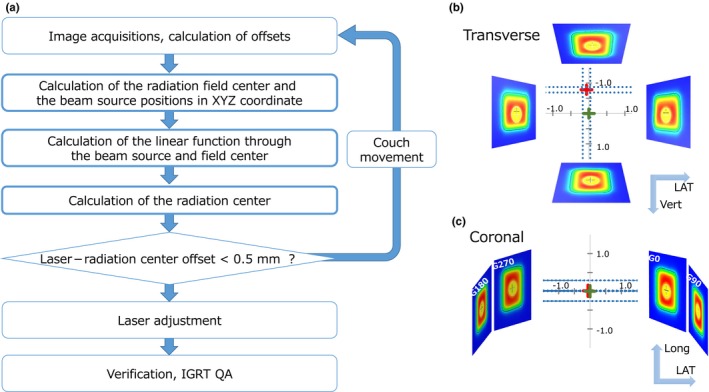
(a) A flowchart of the virtual starshot analysis. Boxes with thick frame represent the steps automatically calculated in the software. Schemes of the four‐field virtual starshot analysis for (b) transverse and (c) coronal planes. Transverse plane showing the lateral and vertical offsets of the beams. Coronal plane showing the lateral and longitudinal offsets of the beams relative to the center of the room lasers. IGRT, image‐guided radiotherapy; QA, quality assurance; LAT, lateral; Vert, vertical; Long, longitudinal.

Two treatment plans were generated using a TPS. One plan contains four coplanar beams with gantry angles of 0°, 90°, 180°, and 270°. Another contains eight coplanar beams with 45° of gantry angle intervals and 12 noncoplanar beams. For noncoplanar beams, the couch rotation angles were 45°, 90°, 270°, or 315°. For each couch rotation angle, three gantry angles were tested: 0°, 30°, and 210° or 0°, 150°, and 330° depending on the clearance between the couch basement and the linac head. Figure 3(a) shows a flowchart of the VS analysis procedure. First, the WL test images were acquired, and the *δ*_Beam, Laser_ value was calculated for each image, as described in section 2.B. For coplanar beams, the *δ*_Beam, Laser_ in the horizontal direction represents the beam offset relative to the laser in the lateral and/or vertical directions, depending on the gantry angle. A linear function of each beam path can be calculated from the following two points: (*P1*) the beam position on the EPID projected to the isocenter plane calculated in the previous section and (*P2*) the beam source position 1000 mm above the isocenter. The lateral (*X*) and vertical (*Y*) coordinates of these two points were calculated as follows:

(1) $$P1_{\left(X,Y\right)}=\left(\delta_{x}\cdot \cos\alpha ,-\delta_{x}\cdot \sin\alpha \right)$$

(2) $$P2_{\left(X,Y\right)}=\left(P1_{X}+1000\cdot \sin\alpha ,P1_{Y}+1000\cdot \cos\alpha \right)$$

here, *α* and *δ_x_* represent the gantry angles recorded in the DICOM header and the *δ*_Beam, Laser_ values in the horizontal axis on the EPID, respectively. In these equations, the origin represents the room laser position. Then, a linear function of the beam path line can be calculated for each beam. As illustrated in [Fig. 3(b)], the beam path lines on the transverse plane can be drawn for four coplanar beams. The center points of all beams were calculated by using the least‐squares method, which is similar to a gantry starshot test using film analysis. The center position determined by this VS analysis should represent the radiation isocenter. The *δ*_Beam, Laser_ in the vertical direction represents the beam offset relative to the laser in the longitudinal direction. When drawing the beam path lines on the coronal view by using vertical offset data, we assumed that all lines were parallel. The radiation isocenter position in the longitudinal direction was calculated as the mean of the vertical offset of all coplanar data [Fig. 3(c)]. To use the mean value, the gantry angle of all beams should be arranged symmetrically to the isocenter. The radiation isocenter position, calculated by using VS analysis, represents three‐dimensional offset values relative to the center of the room laser position (*Δ*_Beam, Laser_). The lateral coordinate of the *Δ*_Beam, Laser_ in the coronal view was determined by the lateral shift in transverse view.

**Figure 4:**
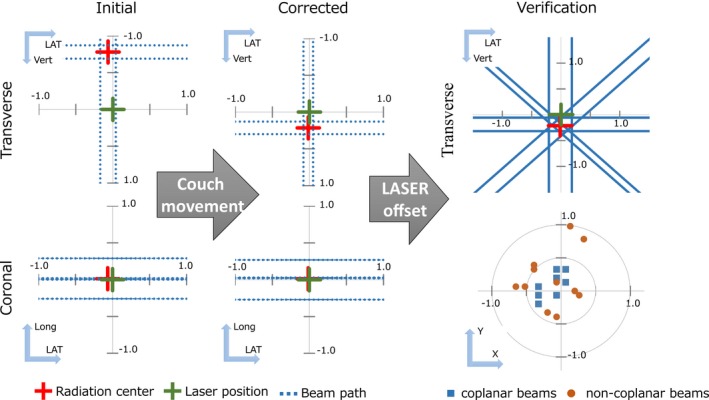
Procedures for adjustment of the room lasers and detailed verifications.

Usually, the sphere for the WL test is fixed on the treatment couch. If the offsets analyzed in the four coplanar beams exceeded a threshold of 0.5 mm for at least one of three‐dimensional axes, the treatment couch was moved to re‐position the sphere to the beam center (Fig. 4). Then, VS analysis with four coplanar beams was repeated to verify whether the corrections were properly performed, and the room lasers were adjusted to the center of the sphere. Subsequently, the WL tests were performed for all beams, including eight coplanar beams and 12 noncoplanar beams. VS analysis was repeated for the coplanar beams because the dynamic conformal arc and volumetric‐modulated arc therapy (VMAT) treatments require accurate beam targeting for various gantry angles. As illustrated in the bottom right of Fig. 4, the *δ*_Beam, Laser_ of all beams was plotted to clearly show the accuracy of the beam targeting for all beams, including noncoplanar beams. If the IGRT images, such as cone‐beam CT (CBCT) and kilovoltage or megavoltage orthogonal 2D images, are acquired after VS analysis, the accuracy of the kV and MV isocenter can also be evaluated. The CBCT image set of the WL sphere, representing the room laser position, was acquired, and the three‐dimensional offset between the origin and center of the sphere was calculated on the treatment console of the linac. In this study, the three‐dimensional offsets between the room laser and imaging center of CBCT (*Δ*_CBCT, Laser_) and between the room laser and radiation center (*Δ*_Beam, CBCT_) were evaluated. Because the couch rotation for noncoplanar image acquisition may slightly affect the position of the sphere, we often conducted IGRT QA after VS analysis, followed by the noncoplanar data collections.

### 2.4 Validation

To validate the technique presented in this study, we performed VS analysis at institution *A–C* with various devices, as illustrated in Fig. 1. Before conducting VS analysis, we verified that the room lasers were aligned within ± 1 mm from the beam isocenter by using a classic gantry‐ and collimator starshot test with Gafchromic^®^ RTQA film (Ashland, Bridgewater, NJ). First, the WL sphere was located at the center of the room laser, and four‐field VS analysis was performed. For validation, the field size of 20 × 20 mm^2^ was used at all institutions to include the displaced sphere inside the beam. The beams used for four‐field VS analysis were generated using Eclipse TPS ver. 11, ver. 13, and ver. 15 (Varian Medical Systems, Palo Alto, CA) at institutions *A*, *B*, and *C*, respectively. Then, the treatment couch on which the WL rod was mounted was translated by 1 mm in the lateral, vertical, and longitudinal directions, and the four‐field VS analysis was repeated. The VS analysis' detectability was evaluated by measuring the difference between the offset values analyzed for the initial and second data. At these institutions, the mechanical accuracy of the three‐dimensional couch motion was checked every month by a read‐out test. A ruler was placed on the treatment couch to fit the scale mark to the room laser, and the couch was moved by a certain distance. The displacement between the ideal scale mark and the room laser was usually ≤ 0.2 mm.

## 3 RESULTS

### 3.1 Validation

Table 2 shows the results of the four‐field VS analysis performed at institutions *A–C*. The *pre* and *post* values represent the *Δ*_Beam, Laser_ values determined by the four‐field VS analysis before and after translating the treatment couch by 1 mm, and *offset* values represent the difference between the two positions. The mean and SD were calculated for the *offset* values of the three institutions. Because the initial VS analyses were performed without the laser adjustments on the day, the results indicate that the room lasers were appropriately controlled. When translating the treatment couch by 1 mm, the differences between the mean ± *SD* offset values analyzed before and after couch movement were 1.01 ± 0.11 mm, 1.05 ± 0.01 mm, and 1.10 ± 0.09 mm for the lateral, vertical, and longitudinal directions, respectively. Given the uncertainties associated with the couch positioning (precision of movements and of the displayed indicators), our results demonstrated that the VS analysis technique could accurately detect the three‐dimensional offset of the sphere from the beam isocenter.

**Table 2:** Offset values from VS analysis with and without couch offsets measured at institutions *A–C*.

Institutions	*A*			*B*			*C*			Mean	SD
	Pre	Post	Offset	Pre	Post	Offset	Pre	Post	Offset		
LAT [mm]	0.04	−1.1	1.14	0.35	−0.58	0.93	−0.15	−1.11	0.96	1.01	0.11
Vert [mm]	0.13	−0.91	1.04	−0.09	−1.13	1.04	−0.22	−1.3	1.08	1.05	0.02
Long [mm]	−0.07	−1.2	1.13	−0.16	−1.16	1.00	0.03	−1.15	1.18	1.10	0.09

Abbreviations LAT, lateral; Vert, vertical; Long, longitudinal; SD, standard deviation.

### 3.2 Long‐term analysis

**Figure 5:**
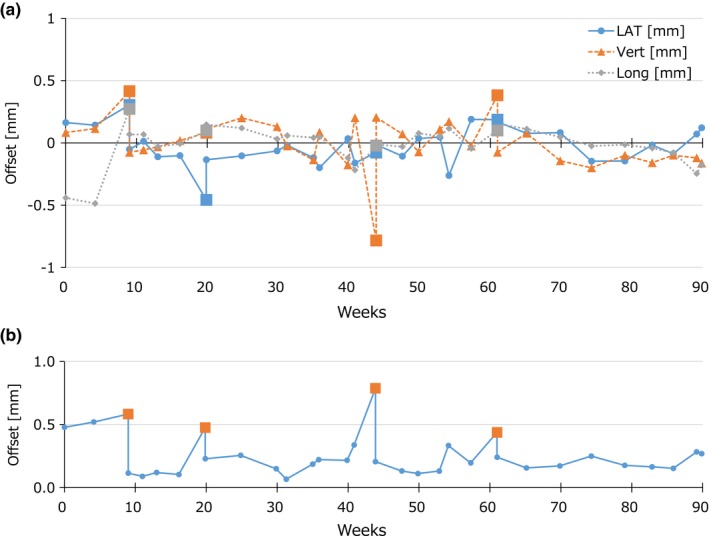
(a) The *Δ*_Beam, Laser_ values along the lateral (LAT), vertical (Vert), and longitudinal (Long) axes from the four‐beam virtual starshot analysis at institution *A*. The horizontal axis represents the weeks from 25 November 2015, the initial measurement day. The square plots represent data points where the room lasers were adjusted to the *Δ*_Beam, Laser_ values. (b) The absolute offset values calculated for *Δ*_Beam, Laser_ values along the three axes from the four‐beam virtual starshot analysis. The horizontal axis represents the weeks from 25 November 2015, the initial measurement day. The square plots represent the data points where the room lasers were adjusted to the *Δ*_Beam, Laser_ values. LAT, lateral; Vert, vertical; Long, longitudinal.

Institution *A* was built in April 2014 and began providing radiotherapy in July 2014. At this institution, VS analysis has been performed monthly and before brain SRT treatments from November 2015. Figure 5(a) shows the *Δ*_Beam, Laser_ values from the four‐field VS analysis. Figure 5(b) shows the three‐dimensional offset between the radiation isocenter and the laser position analyzed by performing the four‐field tests. Square plots represent the tests before adjusting the laser positions. Although the lasers were not adjusted in the first 2 months for observation, the laser alignment was appropriately managed with three‐dimensional accuracy within ± 0.5 mm after the initial adjustments of the laser.

**Figure 6:**
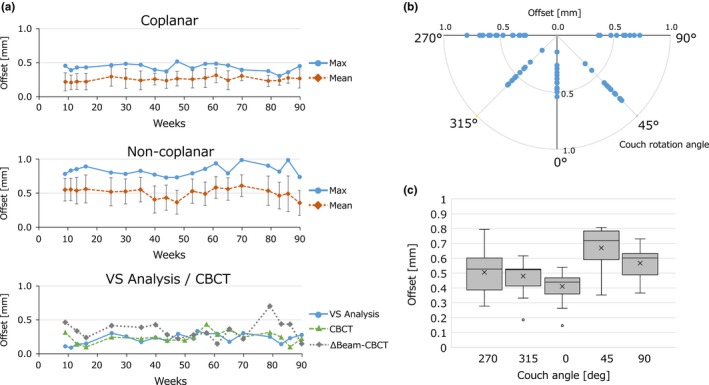
(a) Results of the eight coplanar (Coplanar) beams (upper row) and 12 noncoplanar (Noncoplanar) beams (middle row) collected at institution *A*. Mean and maximum *δ*_Beam, Laser_ are plotted against the weeks from the initial measurement day. Bars represent the standard deviations of the 8 or 12 beams, respectively. The *Δ*_Beam, Laser_ values analyzed for the eight coplanar beams, *Δ*_CBCT, Laser_, and *Δ*_Beam, CBCT_ (VS Analysis/ CBCT) are plotted (lower row). (b) The offset values measured with 0° gantry angle collected at institution *A* plotted against the couch rotation angle. The values of 0°, 45°, 90°, 270°, and 315° represent the couch rotation angle. (c) Distribution of the offset values of institution a measured with 0° gantry angle. Boxes represent the median and range of 25th–75th percentile. Whiskers represent the minimum–maximum range. Crosses and points represent the mean and outliers, respectively.

In [Fig. 6(a)], the absolute *δ*_Beam, Laser_ values analyzed for the eight coplanar and 12 noncoplanar beams measured at institution *A* are shown. The values were calculated as the root mean square of the horizontal and vertical *δ*_Beam, Laser_ values. This analysis started 2 months after starting the four‐field test. The coplanar beams showed that the maximum *δ*_Beam, Laser_ was within 0.6 mm for the entire period. The noncoplanar beams showed slightly larger *δ*_Beam, Laser_ values than those of the coplanar beams. However, the maximum values were within 1 mm for all terms. The maximum offset values exceeded 0.98 mm twice, and the gantry (couch) angles of these two data points were 150° (270°) and 210° (90°). In the bottom of [Fig. 6(a)], the *Δ*_Beam, Laser_ values calculated by VS analysis for the eight coplanar beams are plotted. The three‐dimensional offsets between the room laser and imaging center of the CBCT (*Δ*_CBCT, Laser_), and between the room laser and radiation center (*Δ*_Beam, CBCT_), were also plotted. The *Δ*_Beam, CBCT_ was 0.66 mm at one data point and < 0.5 mm for all other measurements. We also analyzed the noncoplanar data acquired with the 0° gantry angle. The distribution of the offset values measured at the institution *A* is shown in Figs. 6(b) and 6(c). Interestingly, the variations were large for the couch angle of 45°.

**Figure 7:**
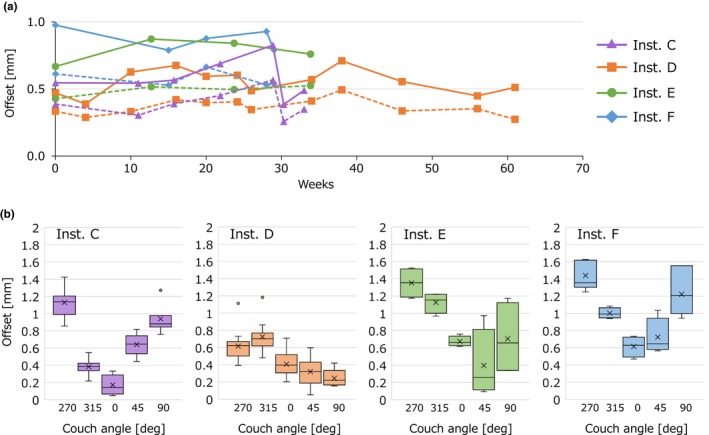
(a) Absolute offset values of the radiation isocenter from the laser center measured at institution *C*–*F* by the virtual starshot technique. Solid and dash lines represent the maximum and mean of the offset values, respectively. (b) Distribution of the offset values measured with 0° gantry angle. Boxes represent the median and range of 25th–75th percentile. Whiskers represent the minimum–maximum range. Crosses and points represent the mean and outliers, respectively. Inst, institution; deg, degree of couch angle.

Figure 7(a) also shows the long‐term analysis of the absolute *δ*_Beam, Laser_ values for institutions *C*, *D*, *E*, and *F*. For all four institutions, the maximum offset was within 1 mm. Figure 7(b) shows the offset of the noncoplanar data acquired with the 0° gantry angle. Variety of couch angle dependencies of the offset values was observed, although the institutions *D* and *E* used the same model of the linacs. These data indicate that the characteristics of the couch rotation may be different even for the same equipment, probably due to the manufacturing and installation.

## 4 DISCUSSION

In this study, we reported a technique to determine the radiation isocenter position using VS analysis. We also assessed the feasibility of VS analysis for adjusting the room lasers projecting to the radiation isocenter by using long‐term data. The technique presented in this study is simple, quantitative, free of inter‐observer variation, and free of uncertainties because of manual processes such as marking for film‐based starshot tests. Image acquisitions were performed on the treatment plans of a virtual patient for QA. Therefore, irradiations and DICOM export can be easily performed. Because we checked the submillimeter accuracy of the couch movement of the TrueBeam at institutions *A–C*, the couch offsets after four‐field VS analysis can be digitally and remotely conducted. Therefore, the procedure, including the WL phantom setup, irradiation of the four‐field beams, DICOM export, VS analysis, couch offset, re‐irradiation of the four‐field beams, and second VS analysis can be completed within 20 min, although the verification beam irradiation takes a little longer because of the couch rotations for noncoplanar beams.

The long‐term data of institution *A* showed that our technique achieved isocenter localization accuracy within 0.6 mm and 1.0 mm for the coplanar and noncoplanar beams, respectively. At institution *A*, we also performed IGRT QA, including orthogonal MV imaging, orthogonal kV imaging, and CBCT, and checked the accuracy within ± 1 mm. Because the TrueBeam STx of institution *A* equips an ExacTrac system (BrainLAB, Munich, Germany),^16^, ^17^ four‐field VS analysis has also been used to calibrate the ExacTrac system. The calibration of the ExacTrac system includes two steps: (a) calibration of the light‐emitting diode (LED) camera using a phantom with LED‐reflecting markers positioned based on the room lasers, and (b) calibration of the X‐ray imaging system using another phantom positioned based on the LED camera system. In addition, the imaging center coordinates calibrated by these procedures can be adjusted to the radiation isocenter defined by the WL sphere. For this adjustment, the WL sphere should be precisely located at the radiation isocenter. With four‐field VS analysis, the WL sphere can be positioned accurately in a short time.

The WL test results are affected by various factors, including gravity which may cause the gantry head sag and displacements of the MLC leaves and carriage, misalignment of the gantry and collimator rotation axes, and mechanical aging degradation. Measurements without considering these effects may include systematic errors. For example, a film‐based collimator starshot measured using a gantry angle of 0° will not include the gantry sagging effects. Du et al.^9^ investigated the effects of the gantry and collimation angle selection used for WL tests and reported that systematic errors arose when using inappropriate angles affected by the linac imperfections, such as gantry sag, gravitational effects, and collimator misalignment. For collimator angles, they reported that gravity effects on the MLC leaves and carriages of 0.17 (0.10–0.28) mm were largest with gantry angles of 90° and 270°. In this study, the collimator angle was 90° for all beams to avoid the gravity effects with gantry rotations. Because the jaw field size was slightly larger than the MLC field size, the center and width of the coplanar beams on the transverse plane were defined by the four‐times of the MLC leaf width, which results in very small uncertainties. To compensate for the uncertainties due to the tongue‐and‐groove effects, both 90° and 270° should be used although the measurement time will double. For VS analysis using coplanar beams, the gantry angles must be equally distributed because the center position was calculated using the least‐squares method. In this study, both four‐field and eight‐field VS analyses used equally distributed gantry angles. Therefore, the results were expected to consider gantry sagging and misalignment of the gantry rotation axis. The gantry and couch angle of the noncoplanar beams did not need to be equally distributed because the values were not used to calculate the radiation isocenter position.

The technique presented in this study includes the following limitations. First, this method evaluates only the combination of gantry and couch angles with which the images can be acquired using EPID. For SRS and SRT for brain metastases, the dynamic conformal arc or VMAT beams are often used with couch rotations. In many such cases, however, the EPID cannot be used because the panel collides with the couch. In this study, we tested the gantry angles of 0°, 30°, and 150° for a 270° couch angle and 0°, 210°, and 330° for a couch angle of 90°. Therefore, we partly checked the noncoplanar situations. Second, the WL sphere was remotely moved using the TrueBeam couch movements after the initial four‐field VS analysis. According to the vendor‐provided specification, the spatial translational accuracy of the treatment couch is ≤ 0.5 mm, but it appeared to be ≤ 0.2 mm in our experience of a monthly mechanical QA. However, such uncertainty will still be smaller than that of the manual adjustments of the sphere position. If the sphere is located at an inappropriate position, it can be detected by the second four‐field VS analysis. In our method, the room laser position was defined as the centroid of the sphere. Because the WL sphere is positioned manually by the staff, the initial room laser position of the analysis may be uncertain. If repositioning is conducted after VS analysis, the final position of the sphere will not remain uncertain, although manual adjustment of the room laser will result in additional uncertainty. These will depend on the WL test devices and on the flexibility of the laser adjustments. Third, the analysis of our WL tests was not fully automated. This algorithm requires manually setting the initial position around the sphere center because the software was designed not only for EPID but also for starshot and WL tests using radiochromic film. This manual process was needed because the optimal threshold for detecting the sphere may vary depending on the sphere size and material. For example, institution C used a hand‐made WL rod with a 3‐mm steel sphere whose radiopacity was lower than that of the tungsten sphere used at other institutions. However, our algorithm successfully analyzed the WL tests for all three spheres. Because the effects of the manual process on the results were negligible (<0.1 mm), all images usually can be simultaneously analyzed within a few seconds by using the same initial position selected for one of the images. At institution *C*, however, we created another hand‐made WL rod with a 5‐mm tungsten sphere and obtained clearer images (data not shown). Creating a hand‐made WL rod and a sphere with highly radiopaque material will provide images with better contrast.

## 5 CONCLUSION

VS analysis was found to be a simple and accurate technique for localization of the radiation isocenter position. The method was validated with sufficient accuracy at three institutions which used different WL spheres and equipment. Long‐term analysis of multiple institutions showed that the technique was feasible for alignment of room lasers and for verification of noncoplanar irradiation and IGRT accuracy.

## CONFLICT OF INTEREST

The authors YA and HS developed Akilles RT, which is a software for QA and research purposes and is currently a commercial software.
